# Are tactile function and body awareness of the foot related to motor outcomes in children with upper motor neuron lesions?

**DOI:** 10.3389/fresc.2024.1348327

**Published:** 2024-03-01

**Authors:** Petra Marsico, Lea Meier, Marietta L. van der Linden, Thomas H. Mercer, Hubertus J. A. van Hedel

**Affiliations:** ^1^ Research Department, Swiss Children’s Rehab, University Children’s Hospital Zurich, Affoltern am Albis, Switzerland; ^2^ Children’s Research Center CRC, University Children’s Hospital Zurich, University of Zurich, Zurich, Switzerland; ^3^ Centre for Health, Activity and Rehabilitation Research, Queen Margaret University, Edinburgh, Scotland

**Keywords:** somatosensory function, monofilaments, cerebral palsy, pediatric stroke, gait, lower limb selective voluntary motor control

## Abstract

**Introduction:**

Somatosensory function can be reduced in children with Upper Motor Neuron (UMN) lesions. Therefore, we investigated relationships between somatosensory functions of the foot and motor outcomes in children with UMN lesions.

**Method:**

In this cross-sectional study, we assessed the Tactile Threshold (TT) with monofilaments and body awareness with Tactile Localisation Tasks for spatial-related action (TLT_action_) and structural-related perception (TLT_perception_) body representation at the foot sole. Furthermore, we assessed four motor outcomes: the Selective Control Assessment of the Lower Extremity (SCALE), the modified Timed Up and Go test (mTUG), the Gillette Functional Assessment Questionnaire (FAQ), and the Functional Mobility Scale (FMS). Spearman's correlations (*ρ*) were applied to assess relationships between the somatosensory function of the foot sole and the applied motor outcomes.

**Results:**

Thirty-five children with UMN lesions, on average 11.7 ± 3.4 years old, participated. TLT_perception_ correlated significantly with all lower limb motor outcomes (|*ρ*|=0.36–0.57; *p *< 0.05), but TLT_action_ (|*ρ*|=0.00–0.27; *p *= 0.15–0.97, and TT did not (|*ρ*|=0.01–0.83; *p *= 0.73–0.94). TLT_perception_ correlated strongly with the Gross Motor Function Classification System (|*ρ*|=0.62; *p *= 0.001) in children with cerebral palsy (*n* = 24).

**Discussion:**

Assessing structural body representation of the foot sole should be considered when addressing lower limb motor impairments, including gait, in children with upper motor neuron lesions. Our results suggest that the assessment of tactile function and spatial body representation may be less related to lower limb motor function.

## 1 Introduction

Children with lesions of the upper motor neuron (UMN) show impairments in lower limb somatosensory functions in addition to impairments in motor function (1–5). In a recent Delphi study, experts agreed that the somatosensory function modalities of tactile function (exteroception), spatial and structural body representation (body awareness), and joint movement, joint position, and dynamic position sense (proprioception) are relevant when assessing somatosensory impairments in children with UMN lesions (6). Body representation includes the localising of touch stimuli by pointing directly at the limbs (spatial) or a visual illustration of the corresponding limbs (structural), while proprioception is the sense of movement and position of limbs in space (7).

Body representation can be assessed with Tactile Localisation Tasks (TLTs). In the TLT, participants point directly to their limbs or a visual representation of the corresponding limbs to localise tactile stimuli. Both tasks reveal facets of body representation, reflecting body awareness. The ability to pinpoint a tactile stimulus on the limbs contributes to the spatial body representation associated with actions. Conversely, the localisation of touch on an image is considered a structural body representation, linked to perception - specifically, the knowledge and awareness of the position of body parts (8). Indeed, studies after stroke focusing on the upper limb have shown that central processing differs when patients point to location on their own body (TLT_action_) compared to locating the body part on an illustration (TLT_perception_) (9, 10). In TLT_perception_, additional brain areas, particularly in the anterior insula, are active in processing body representation (8).

In a study by Hoon and colleagues of 28 children with leukomalacia due to preterm birth (aged 1.5–13 years), over 90% of the children had abnormalities in their sensory pathways, as observed on diffusion tensor imaging (11). More interestingly, the severity of damage in the posterior thalamic tracts was significantly related to the severity of sensory and motor impairment (12). When focusing on lower limb tactile function and body awareness, two studies reported that children with unilateral and bilateral Cerebral Palsy (CP) had significantly lower tactile function measured at the foot sole than typically developing peers (4, 5). In another study, 40 children with UMN lesions (CP (*n* = 26), acquired brain lesions (*n* = 7), other diagnoses such as hydrocephalus, congenital ataxia (*n* = 7)) had significantly lower tactile function and body awareness assessed at the soles of their feet compared to 40 typically developing peers (3).

However, in general, impairments in somatosensory functions are not routinely assessed in clinical practice (13) because, among other reasons, most assessments do not fulfill the key requirements for somatosensory assessments, recently identified by a group of experts: child-friendliness, practicality, and relevance to motor function (6). Accordingly, impairments in somatosensory functions are often not identified nor taken into account in therapy programs. For instance, recent outcome measures to test tactile function do not include a detailed test protocol, which hinders a standardised application (4, 14), and outcome measures assessing body representation require specialised equipment and software not available or suitable for use in routine clinical practice (15).

Regarding the relevance of somatosensory aspects to motor function, studies investigating the relationship between lower limb impairments in tactile function and body awareness and motor outcome show conflicting results. A moderate relationship was observed between tactile function, assessed with monofilaments to assess the tactile threshold (TT) on the foot and gait quality in 15 children with bilateral spastic CP (4). In contrast, in the same study, such a relationship was absent in 15 children with unilateral spastic CP (4). Zarkou and colleagues observed a moderate relationship between tactile function and walking endurance quantified by the 6-minute walk test in 10 participants with CP (14). However, in the same study, tactile function did not correlate with postural control and balance (14). One study investigated associations between body awareness of the upper and lower limb and motor function in children with motor deficits (16). The authors assessed structural representation of the body in 18 children with motor deficits (nine with CP, five with autism spectrum disorder, three with intellectual disability, and one with attention deficit hyperactivity disorder) using a tactile localisation task for the lower limbs and toes. They found significant correlations between lower limb structural body representation and one-leg standing (*r* = 0.70; *p *< 0.01), one-leg hopping (*r* = 0.69; *p *< 0.01), and the total Gross Motor Function Measure (GMFM; *r* = 0.80; *p *< 0.001. In contrast, they did not find significant correlations between the tactile localisation of the toes and motor activities (16).

No study has investigated the relationships between tactile function and body awareness of the lower extremities with motor outcomes in children with UMN. Therefore, the current study aimed to investigate the relationships between tactile function, spatial and structural body representation, and lower limb motor outcomes in children with UMN lesions.

Based on previous studies (4, 14, 16), we hypothesised that a modest relationship between somatosensory function and lower limb motor outcomes would exist. Furthermore, we expected to observe a moderate negative correlation between tactile function and body awareness and the Gross Motor Function Classification System (GMFCS) in those children diagnosed with CP.

## 2 Materials and methods

### 2.1 Study design

We used a cross-sectional, observational study design.

### 2.2 Participants

We recruited 35 children and youths from the in- and outpatient setting of the Swiss Children's Rehab Centre of the University Children's Hospital Zurich with the following. Inclusion criteria: neuromotor impairments due to UMN lesions (including diagnoses such as CP, other congenital brain lesions, and acquired brain injury), age five to 19 years, ability to lie 15 min in a prone position, ability to bear weight on the legs, standing and walking with or without a walking aid or some support for short distances. Exclusion criteria were having undergone surgery with involvement of the lower limbs within the last six months, botulinum toxin injection in lower limbs within the previous six months, unable to communicate pain or discomfort (verbally or nonverbally), noncompliance, and being unable to follow simple short instructions.

We described the participants by sex, body weight and height, diagnoses, medication, and aids (e.g., walking aids). In addition, we recorded the GMFCS level for children with CP to classify the functional abilities and limitations in the gross motor function (Level I = slight motor limitations, and Level V = severe motor limitations) (17).

All children and youths agreed verbally to participate, and parents and adolescents aged 14 years and older also signed the informed consent form. The study was approved by the Cantonal Ethics Committee of Zurich (BASEC-Nr. PB_2016-01843) and followed the good clinical practice guidelines. We aimed to collect data from at least 35 participants to reach the requirement for correlation analysis to detect a correlation coefficient of 0.5 with an alpha of 0.05 and a power of 80% (18).

### 2.3 Measurements

Two physiotherapists (first and second author) with over ten years of experience in neuro-pediatric rehabilitation applied the somatosensory assessments. For a previous study, the two testers had practiced the somatosensory tests and used these in 40 children without neurological impairment (3). The first author uses the SCALE and the mTUG regularly in everyday clinical practice and has also utilised these in previous studies with children with neurological impairment (19, 20).

**Figure 1:**
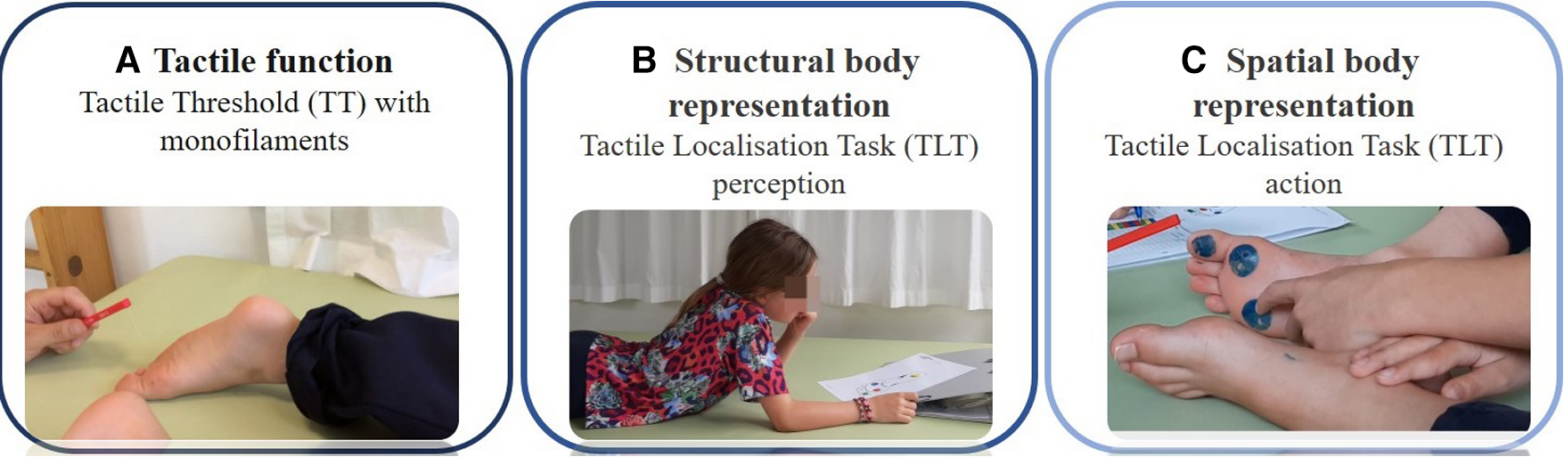
Somatosensory function assessments. Legend: (**A**) Tactile function assessed with monofilament as part of exteroception (How fine tactile inputs can they detect?); (**B**) Structural body representation assessed with a Tactile Localisation Task (TLT) perception (Can they localise the tactile input on an illustration of the body?); and (**C**) Spatial body representation assessed with a Tactile Localisation Task (TLT) action, as part of body awareness (Can they localise the tactile input on their body?).

The assessments took place in a quiet therapy room and lasted no longer than one hour. The assessments were carried out in the following order: First, the TT test to assess tactile function, followed by the two tests for TLT: first, the TLT for perception (TLT_perception_), where the children had to identify a tactile input by pointing to the illustration of a foot sole. Second was the TLT action (TLT_action_) test, where the children had to identify and localise a tactile input and point directly at the location on their foot soles (Figure 1). The less affected leg was assessed first, followed by the more affected leg. The more affected leg was identified from the medical records of each participant as the leg exhibiting lower selective motor control. Finally, the first author scored the Selective Control Assessment of the Lower Extremity (SCALE), again, first for the more affected leg, followed by the less affected one. The same therapists assessed the mTUG test.

### 2.4 Somatosensory function measures

The measurement protocol was described in our previous study (3), in which we used these three somatosensory tests in 40 children with UMN lesions and 40 typically developed peers. The tests showed reasonable practicability (98% participation rate, mean duration of the three tests' application was 18 min), could differentiate well between children with and without UMN lesions, and showed high convergent validity between TT and TLT action (*ρ* = 0.71, *p *< 0.001), and TLT_action_ and TLT_perception_ (*ρ* = 0.66, *p *< 0.001), and fair between TT and TLT_perception_ (*ρ* = 0.31, *p *= 0.01). The inter-rater reliability analyses for the sum scores showed almost perfect agreement for the TT expressed with quadrated weighted kappas (*κ*_QW_ more affected leg 0.86; less affected leg 0.81), substantial agreement for TLT_action_ (*κ*_QW_ more affected leg 0.76; less affected leg 0.63), and almost perfect agreement for TLT_perception_ (*κ*_QW_ more affected leg 0.88; less affected leg 0.74).

For the TT, we tested four areas of each foot sole with Semmes-Weinstein monofilaments from the foot set of Baseline® Tactile™ (Colorado, United States). The four areas were the big toe, the first and fifth metatarsal head, and the heel. The monofilaments were applied perpendicularly for a maximum of two seconds on the skin in random order on the location of the foot sole. We used five monofilaments (0.4 g, 2 g, 4 g, 10 g, 300 g). For the monofilaments, we started with the 4 g monofilament. If the child could feel two of three attempts, the tester used the next thinner monofilament until the child could identify the thinnest monofilament for each tested area, defined as Tactile Threshold (TT). The child lay supine and could not see the assessor's movements when applying the monofilaments. The TT was expressed at an ordinal scale from 0 to 5, with 0 indicating no detection of light touch (i.e., no detection of the 300 g monofilament) and 5 reflecting the thinnest monofilament (0.4 g, which means normal light touch detection). Finally, we calculated a sum score for the more and less affected side, by summing the scores of the four areas; hence, each sum score could be between 0 and 20 (3).

To evaluate both spatial body representation (TLT_action_) and structural body representations (TLT_perception_), the examiner randomly applied the 10 g monofilament to the same four areas assessed with the TT on the sole. Each area was tested three times. If a child could not feel the 10 g monofilament, the assessor used the next bigger monofilament. For the TLT_action_, the children were blindfolded, so they could not see their feet. The children were sitting comfortably, and the investigator asked them to point to the area of the foot sole where they felt the tactile input. For the TLT_perception_, the children were lying supine, so they could not see their feet. After the investigator had touched the sole, they had to identify the place of tactile input on an illustration of the feet (3). The child was awarded one point per defined area for each correct identification. The maximum value per area was 3 (i.e., 3 out of 3 trials). The sum scores for the more and the less affected sides were calculated by summing the scores for each of the four areas, which means possible scores ranging from 0 to 12 per foot. We used these scores for further calculations.

### 2.5 Lower limb motor outcomes

According to the international classification of functioning, disability and health (ICF), we conducted four lower limb motor outcome assessments (21). The domain body function was assessed by the SCALE, which assesses selective voluntary motor control of the legs. The SCALE measures and categorises selective motor control of the hip, knee, ankle, subtalar, and toe joints as normal (score 2), impaired (score 1), or unable (score 0) (20, 22). The SCALE showed high reliability and validity in children with CP (20). The mTUG covers the ICF domain activity by assessing walking capacity, including balance components. The time needed to stand up from a chair, walk three meters to a target, touch it, turn around, walk back to the chair, and sit down was measured. The children could use their everyday assistive devices. Shorter times indicate better functioning while running is not permitted. We performed the mTUG twice and included the average value of the two trials in the analyses. The mTUG is reliable and valid in children with CP (23). The Functional Mobility Scale (FMS) and the Gillette Functional Assessment Questionnaire (FAQ) describe the performance level of mobility in daily life (ICF domain activity. The treating physiotherapist filled out the FAQ. For the FMS, the parents or the participants' physiotherapists rated the walking ability at three specific distances (5 metres, 50 metres, and 500 metres) in everyday situations. Each distance was scored between 1 (using a wheelchair) and 6 (independent walking on any surface (24, 25). The FAQ includes a walking scale encompassing a range of walking abilities from non-ambulatory to ambulatory in all community settings and terrains (24, 26). A score of one describes the lowest level as “cannot take any steps at all” and ten, the highest level, “walks, runs, and climbs on level and uneven terrain and does stairs without difficulty or assistance. Is typically able to keep up with peers.”

### 2.6 Statistical analysis

The statistical analyses were conducted using SPSS version 27 (IBM SPSS Statistics, Chicago, IL). We used the Wilcoxon test to investigate differences between the somatosensory function outcomes of the more and less affected legs. We quantified the relationships between the somatosensory and the lower limb motor outcomes using Spearman's correlation coefficients (*ρ*). We used the following benchmarks for the degree of correlation; little (poor) relationship (0–0.24), a fair degree of relationship (0.25–0.49), a moderate to good relationship (0.50–0.74), and a very good to excellent relationship (0.75–1.00) (27). For all analyses, alpha was set at *p*-value = 0.05.

## 3 Results

Thirty-five children with UMN lesions and a mean age of 11.7 years (SD 3.4 years, range 5–19 years) participated (for characteristics, Table 1).

**Table 1:** Participant's characteristics of the children with UMN lesions (*n* = 35).

Variables	Characteristics		Number (*n*)
Age groups	5 to ≤10 years		10
	10 to ≤14 years		18
	14 to ≤19 years		7
Sex	Girls		22
	Boys		13
Topography of the motor disorder	Unilateral		15
	Bilateral		20
More affected leg	Right leg		22
	Left leg		13
Medication^a^	No medication		20
	Pain medication		2
	Anti-spastic		4
	Anti-epileptics		5
	Other medication^a^		4
Diagnosis^b^	Cerebral palsy		25
	GMFCS	Level I	11
		Level II	4
		Level III	4
		Level IV	5
		Level V	1
	Stroke		6
	Traumatic brain injury		1
	Congenital ataxia		3
Type of muscle tone	Spastic		18
	Ataxia		4
	Mixed tone		11
	No atypical tone		2

^a^ Other medication includes medication against allergic reactions or nausea.

^b^ 28 children had a congenital brain lesion, six an acquired brain lesion, and one child had both diagnoses.

One young child could not perform the TLT_action_ and TLT_perception_ tests (5.2 years, GMFCS level III). Further, due to their motor impairment, four children with GMFCS level IV and the only participant classified with GMFCS level V could not participate in the TLT_action_. Four children could not complete the mTUG either, as they did not have the motor ability to perform the mTUG independently. Therefore, the number of participants varied between 29 and 35 in the correlation analyses.

### 3.1 Somatosensory function and lower limb motor outcomes

We present the median, interquartile range (IQR), minimum, and maximum values of the sum scores of the tested children in Table 2. The median scores of the TT were high and only one point below the maximum. The IQR varied more for the TLT_perception_ than for the TLT_action_, and the TT. For the TT test, 51% of the children reached the maximum, meaning they had a normal TT for the more and less affected side. For the TLT_action_, 41% reached the maximum points for the more and 55% for the less affected side, and for the TLT_perception_, 38% of the children achieved the maximum score for both legs.

**Table 2:** Participants’ results from the somatosensory function and lower limb motor outcomes, differences between the more and less affected leg.

Measures	Participants (*n*)	More affected leg		Less affected leg	
		Median (IQR)	Min/Max	Median (IQR)	Min/Max
TT: *total score* (*0–20*)	35	20.0 (16.5–20.0)	5.0/20.0	20.0 (19.0–20.0)	11.0/20.0
TLT_action_: *total score (0–16)*	29	11.0 (8.0–12.0)	2.0/12.0	11.0 (9.0–12.0)	2.0/12.0
TLT_perception_: *total score (0–16)*	34	10.5 (6.0–12.0)	2.0/12.0	11.0 (6.3–12.0)	2.0/12.0
SCALE: *total score (0–10)*	35	4.0 (2.0–6.50)	0.0/9.0	7.0 (4.0–8.0)	0.0/10.0
		Median (IQR)		Min/Max	
mTUG: *time (s)*	31	6.4 (5.4–8.2)		3.6/47.4	
Total FMS: *score (3–18)*	35	18.0 (5.5–18.0)		3.0/18.0	
5-meter *(1–6)*		6.0 (2.0–6.0)		1.0/6.0	
50-meter *(1–6)*		6.0 (2.0–6.0)		1.0/6.0	
500-meter *(1–6)*		6.0 (1.0–6.0)		1.0/6.0	
FAQ *score (1–10)*	35	9 (6–9)		1.0/10.0	

TT, tactile threshold; TLT, tactile localisation task; SCALE, selective control assessment of the lower extremity; mTUG, modified timed up and go; FMS, functional mobility scale; FAQ, Gillette functional assessment scale, IQR, interquartile range.

All children except two had the ability to feel the 10 g monofilament. Therefore, the 10 g could be used to test body representation, except for these two children, where the 300 g monofilament was used to assess TLT. The somatosensory assessments showed no statistically significant differences between the more and less affected leg (*p*-values for TT = 0.12; TLT_action _= 0.64; TLT-_perception _= 0.40 of the Wilcoxon test.

### 3.2 Relationships between somatosensory and lower limb motor outcomes

**Figure 2:**
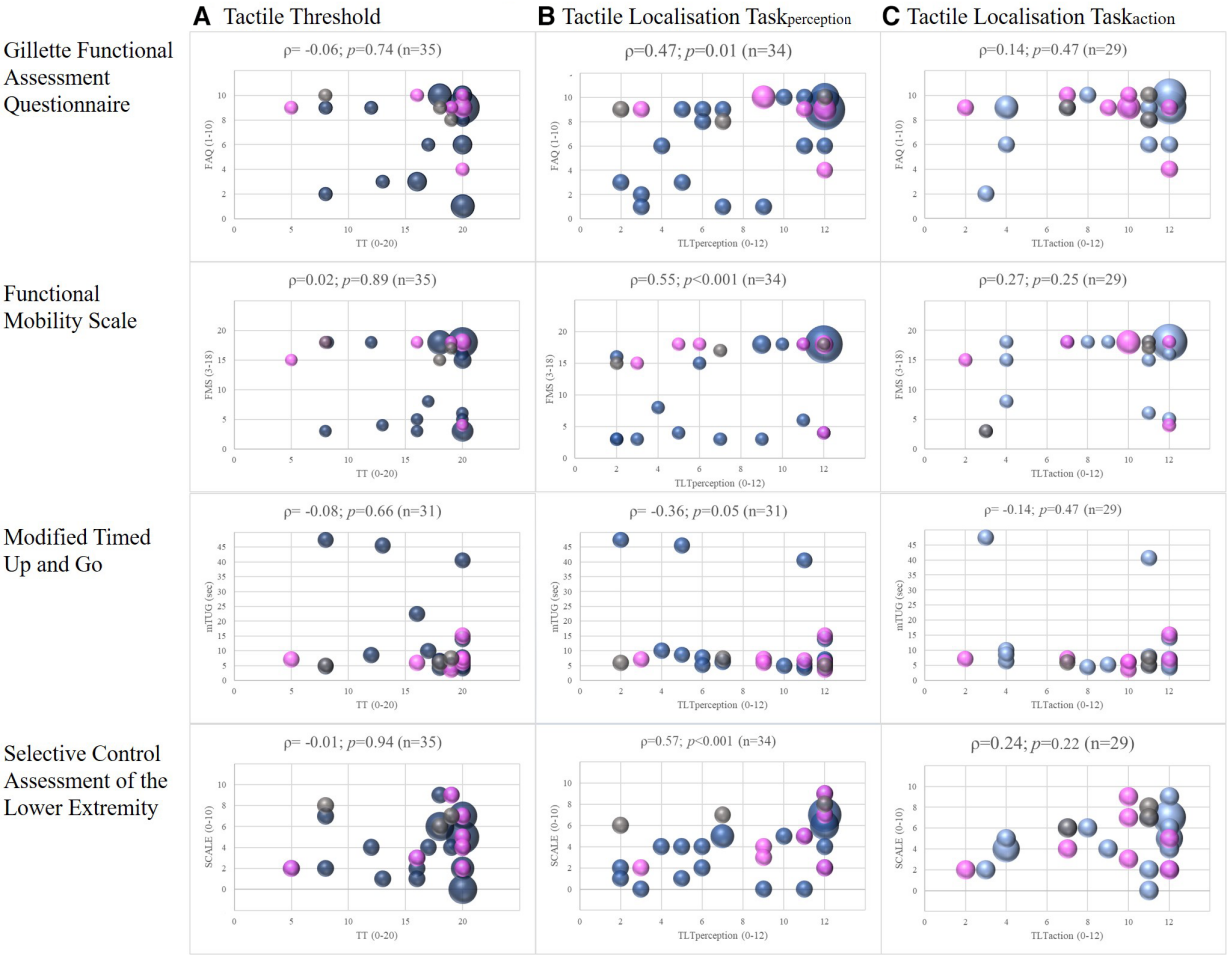
Correlations between somatosensory function and lower limb motor outcomes, with spearman correlations (*ρ*) of the more affected leg. Legend: The colours represent the different diagnosis: blue = children with cerebral palsy; pink = children with acquired brain lesions; grey = children with congenital ataxia. The size of the dots indicates the number of participants (the larger the dots, the more children were pictured).

There was no relationship between the TT and any of the four lower limb motor outcomes for either side, i.e., the more affected leg (Figure 2) and the less affected leg (Supplementary S1). We found a fair degree of relationship between FMS and the TLT_action_ of the more affected side, (|*ρ*| = 0.27) but this was not significant *p* = 0.15) (Figure 2). The other correlations, also for the less affected leg (Supplementary S1), were poor and also insignificant (|*ρ*| < 0.25). In contrast, the TLT_perception_ of the more affected legs correlated moderately to good with the SCALE total scores (|*ρ*| = 0.57; *p* < 0.001) and the FMS (|*ρ*| = 0.55; *p* < 0.001) and fair with the mTUG (|*ρ*| = 0.36; *p* = 0.05) and the FAQ (|*ρ*| = 0.47; *p* = 0.01; Figure 2). The results of the less affected leg were similar to those of the more affected leg (Supplementary S1).

**Figure 3:**
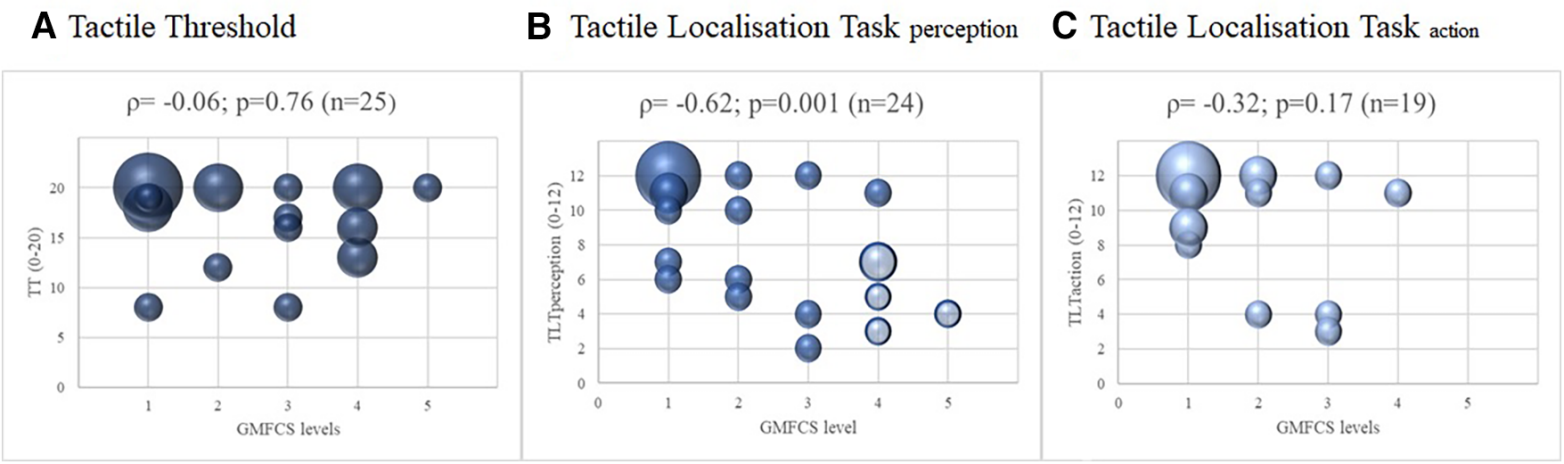
Correlations between somatosensory function and the level of gross motor function classification system of the children with cerebral palsy, with spearman correlations (*ρ*) of the more affected leg. Legend: The four shaded dots in (**B**) Tactile Localisation Task (TLT) perception represent the five children that could not perform the Tactile Localisation Task (TLT) action. The size of the dots indicates the number of participants (the larger the dots, the more children were pictured).

For the subgroup of children diagnosed with CP, we also investigated the relationship between the GMFCS and the three outcomes of somatosensory function. For the TT and TLT scores of the more affected leg, the correlation with the GMFCS was moderate to good for the TLT_perception_ (|*ρ*| = 0.62; *p *= 0.001; Figure 3), fair for TLT_action_ (|*ρ*| = 0.32; *p *= 0.17), and poor for TT (|*ρ*| = 0.06; *p *= 0.76). The results were similar for the less affected leg; the correlations with GMFCS were moderate to good for TLT_perception_, fair for TLT_action_, and poor for TT (Supplementary S2).

## 4 Discussion

We investigated the relationships between several somatosensory functions and lower limb motor outcomes, including gait, to investigate which somatosensory functions might be relevant for lower limb motor function. Indeed, an expert panel considered this an essential requirement for lower limb somatosensory assessments (6). We found a moderate to good relationship between the TLT_perception_ test performed at the foot sole and the lower limb motor outcomes. In contrast, such a relationship was poor for the TT and poor to fair for the TLT_action_. We also analysed the correlations between the somatosensory function measures and the GMFCS level in the sub-sample of children with CP. A moderate to good relationship was found between the TLT_perception_ test and the GMFCS, and a fair but non-significant relationship between the TLT_action_ and the GMFCS. Children with better motor function performed better on both tests. Whereas the TT showed no relationship with GMFCS scores.

Although we detected in a previous study that children with UMN lesions had higher TT than those developing typically (3) we could not identify relationships between the TT and lower selective lower limb control, gait capacity, or gait performance. To our knowledge, only two previous studies investigated the relationship between TT and motor outcomes. Our results are partly in line with those of Zarkou and colleagues (14), who reported in 10 children with CP a fair but non-significant relationship between the TT and balance performance. However, they found a significant relationship between the TT and the 6-minute walking test indicating that a more impaired TT related to a shorter distance walked. In another study, a fair correlation between TT and quality of gait, assessed with the Edinburgh Visual Gait Score, was found in 30 children with CP (4). As we are unaware of any study investigating the relationship between body awareness and motor outcomes in children with acquired UMN lesions, we cannot compare our results for this group of children to the literature.

We can only speculate why our results differ from the literature. While the method to assess the TT was the same in all studies, we had different motor outcomes, which might explain the results. Another argument could be the participant sample included in our study. We included also children with acquired brain lesions, when focusing for those children with CP, GMFCS levels I–IV. In contrast, the other studies included only children with CP levels I–II (4) or I–III (14, 16). However, even when repeating the analyses for children with CP (*n* = 25), we noted a similar low relationship between the TT of the more affected leg and the SCALE (|*ρ*| = 0.03; *p *= 0.89), the FMS (|*ρ*| = 0.07; *p *= 076), and the FAQ (|*ρ*| = 0.07; *p *= 0.75), which confirmed our overall results. The results for the less affected leg were comparatively low. Only the mTUG of the more affected leg correlated slightly more with tactile function (|*ρ*| = 0.30; *p *= 0.18) in the 20 children with CP who could walk. However, in the less affected leg, the correlation was again low (|*ρ*| = 0.19; *p *= 0.40). In conclusion, our results indicate that in children with UMN lesions, TT is not related to lower limb motor function, gait capacity, and gait performance. Furthermore, in the study of Uzun-Akaya and Elbasan (2021), only children with GMFCS levels I and II participated (*n* = 30), while in the study by Zarkou et al., children classified within GMFCS levels I–III were included (*n* = 10) (14). In our study, children classified within GMFCS levels IV and V were also included. These children do not walk or walk only in a therapeutic setting. However, they demonstrated high tactile function (sum scores TT between 14 and 20), indicating their ability to sense the touch of thin monofilaments. This may provide further insight into the limited correlation observed with their motor outcomes.

The correlations between TLT_action_ and the lower limb motor outcomes were also low. Unfortunately, no study has investigated TLT_action_ in children with UMN until now, so we cannot contextualize our results in light of previous findings. The relationship between the GMFCS levels and the TLT_action_ test in the subgroup of children with CP was fair (*n* = 19; *ρ* =* *0.32: *p *= 0.17). Five children with severe motor limitations (GMFCS levels IV and V) could not perform the TLT_action_ test (see Figure 3 and Supplementary S2). Some of these children had poor outcomes localising the area of tactile input on the illustration of the foot (TLT_perception_). If these children had been able to perform the TLT_action_ test and had also performed poorly on this test, this could have strengthened the results of the correlation analyses between TLT_action_ and motor outcomes. As mentioned in the introduction, TLT_action_ assesses a different modality of body awareness. In our study, however, TLT_perception_ shows a stronger relationship with the motor outcomes used in our study than TLT_action_. This may also be due to the selected motor outcome measures.

We found statistically significant correlations between TLT_perception_ of the foot sole and all four lower limb motor outcomes. These results suggest that children with lower scores on TLT_perception_ tend to have poorer selective function, gait capacity, and performance. A neurophysiological explanation for the strong relationship between structural body representation and motor outcomes could be provided by the model of De Haan and Dijkerman (8). They show that structural body representation (TLT_perception_) requires additional brain areas, such as the anterior insula, to process this somatosensory information (8). This could explain why children with lower motor skills, with more complex brain damage, also show more impairments in structural body representation. Indeed, Hoon et al. used diffusion tensor imaging to show that children with CP have impairments in their thalamocortical connections. They speculated that this loss of sensory connections alters the sensorimotor connection to the motor cortex (12). Also, Asano and Morioka found a strong association between lower limb TLT_perception_ (assessed at four points of the proximal and distal parts of the thigh and the lower leg) and single-leg standing, single-leg hopping, and the GMFM in 18 children with motor impairments due to a variety of health conditions (16). In their study, the tactile inputs were applied on four points on the children's thighs and lower legs, and the children had to show the tactile input on an illustration where circles illustrated the four points. They calculated a percent score of the correct answers. In our study, we used a total score ranging from 0 to 12 by tactile inputs on the three areas of the foot sole. Due to the good relationship between TLT_perception_ and lower limb motor function shown in the study by Asano and Morioka (12) and our study, we recommend amalgamating the procedures of the two tests. That primarily involves assessing the thigh, lower leg, and foot sole.

### 4.1 Methodological considerations

In our study, we performed the assessments on the foot sole. However, as mentioned above, Asano and Morioka's (16) study applied the TLT_perception_ at the proximal and distal parts of the thigh and the lower leg. Therefore, we recommend that future studies include various body parts when assessing body awareness.

It is important to note that we only included three somatosensory modalities to investigate the relationship with motor control. Previous studies have shown that other aspects, such as vision, muscle strength, and trunk control, influence gait capacity and performance (28–30). Although the assessments we investigated only captured some related factors, structural body representation moderately correlated with the applied assessments. We suggest that proprioception, another important category of somatosensory function, is also related to selective motor control, gait capacity, and performance. Another aspect to investigate is the association between somatosensory function and quality of movement, as the quality of movement is one aspect that is important for energy-balanced mobility, and enhancing gait quality is often a goal listed by children undergoing gait rehabilitation (28). Therefore, in addition to measures of capacity and performance, future studies should include assessments of quality of movement, such as the Quality Function Measure (QFM) (31). In addition, there should be more focus on how somatosensory functions influence motor control. Studies are also needed to explore potential causal relationships, such as whether training specific somatosensory functions can improve motor control in children with UMN lesions.

Age could have influenced the somatosensory outcomes in our study. For example, spatial body representation continues to develop until the age of seven, and structural body representation until the age of nine (32). However, no significant associations between age and somatosensory outcomes were identified in our study (*ρ* ranging from −0.17 to 0.07, *p *> 0.05). Similarly, post-hoc Mann–Whitney *U*-tests found no statistically significant differences between girls and boys for the TT, TLT_action_, and TLT_perception_.

### 4.2 Study limitations

In our study group, we included 25 children with CP, three with congenital ataxia, and seven with an acquired brain lesion. We could analyse the group of children with CP separately but not the other two groups, as they were too small. Larger groups of children with these diagnoses would be needed to learn more about the specific impairments of somatosensory function and correlations with motor outcomes in these two groups.

Finally, we cannot generalised our results to children with acquired brain lesions in acute or subacute phase. We only included children in a late phase after injury (>6 months; *n* = 7). Similar to adults after brain injury, there could be a higher impairment, especially for TT, in the acute phase after brain injury (see above).

## 5 Conclusion

Our results indicate that structural body representation assessed with a TLT_perception_ assessment on the foot sole relates to lower limb selectivity, gait capacity, and performance. Furthermore, in the subgroup of children with CP, the TLT_perception_ sum scores correlated well and significantly with the GMFCS. Therefore, we recommend assessing the structural body representation of the lower limbs in clinical practice. The close relationship with the motor outcomes may open up new therapeutic options for improving lower limb motor function in these children. While other therapeutical reasons exist to assess impairments in tactile function and spatial body representation, our current results suggest that these outcomes may be less relevant to lower limb motor functioning.

## Data Availability

The original contributions presented in the study are included in the article/Supplementary Material, further inquiries can be directed to the corresponding author.

## Supplementary material

Supplementary Figure S1 Correlations between somatosensory function and lower limb motor outcomes, with Spearman correlations (*ρ*) of the less affected leg. The colours represent the different diagnosis: blue = children with cerebral palsy; pink = children with acquired brain lesions; grey = children with congenital ataxia. The size of the dots indicates the number of participants (the larger the dots, the more children were pictured).

Supplementary Figure S2 Correlations between somatosensory function and the level of gross motor function classification system of the children with cerebral palsy, with spearman correlations (*ρ*) of the less affected leg. The four shaded dots in B) Tactile Localisation Task (TLT) perception represent the children that could not perform the Tactile Localisation Task (TLT) action. The size of the dots indicates the number of participants (the larger the dots, the more children were pictured).
