# Supplementary figures and images for: Are tactile function and body awareness of the foot related to motor outcomes in children with upper motor neuron lesions?
Source: Front Rehabil Sci. 2024 Mar 1;5:1348327. doi: 10.3389/fresc.2024.1348327 (PMC10940356; doi:10.3389/fresc.2024.1348327)

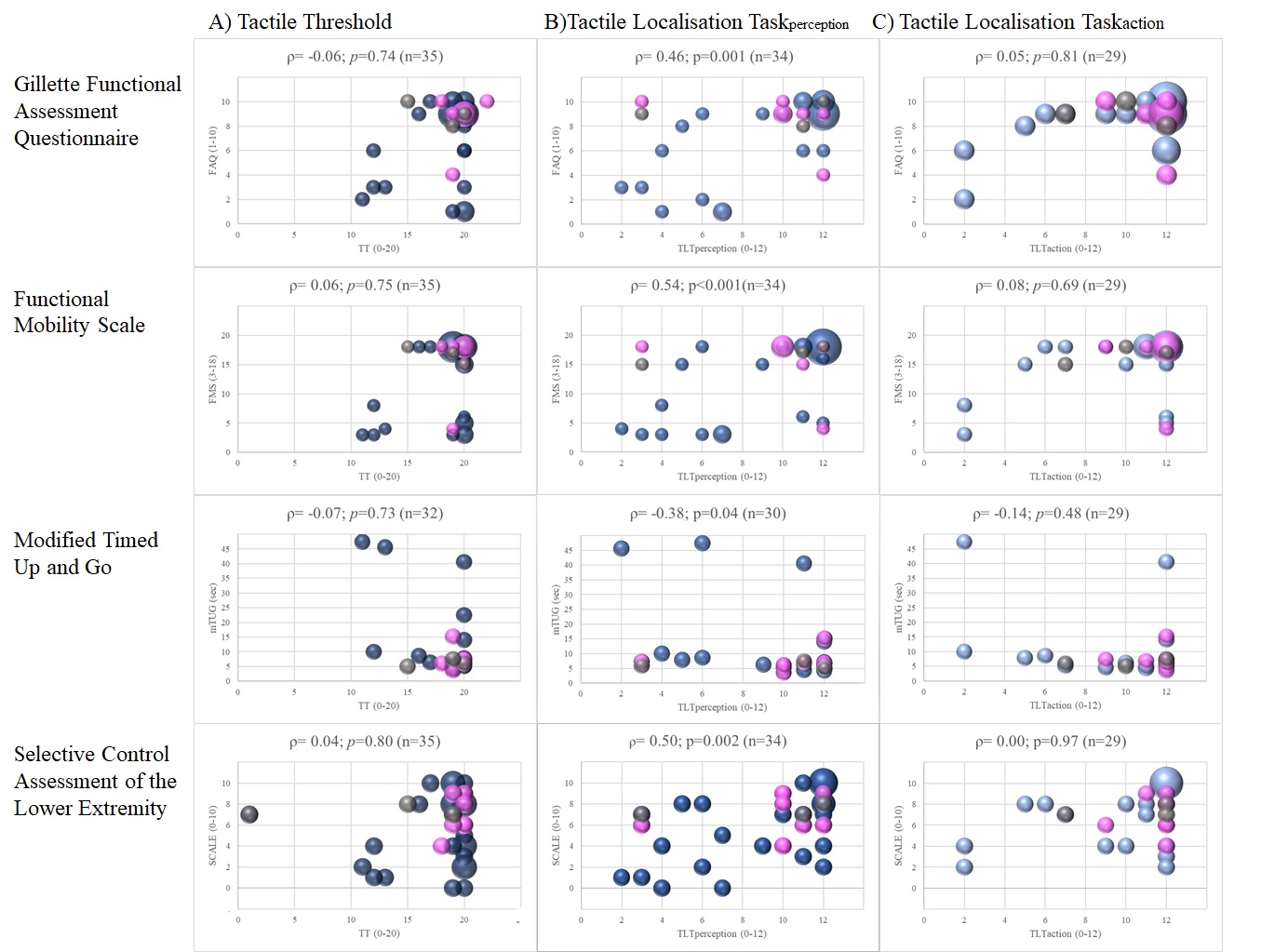

Supplement: Supplementary Figure S1 — Correlations between somatosensory function and lower limb motor outcomes, with Spearman correlations (ρ) of the less affected leg. The colours represent the different diagnosis: blue = children with cerebral palsy; pink = children with acquired brain lesions; grey = children with congenital ataxia. The size of the dots indicates the number of participants (the larger the dots, the more children were pictured). [file Image1.jpeg]

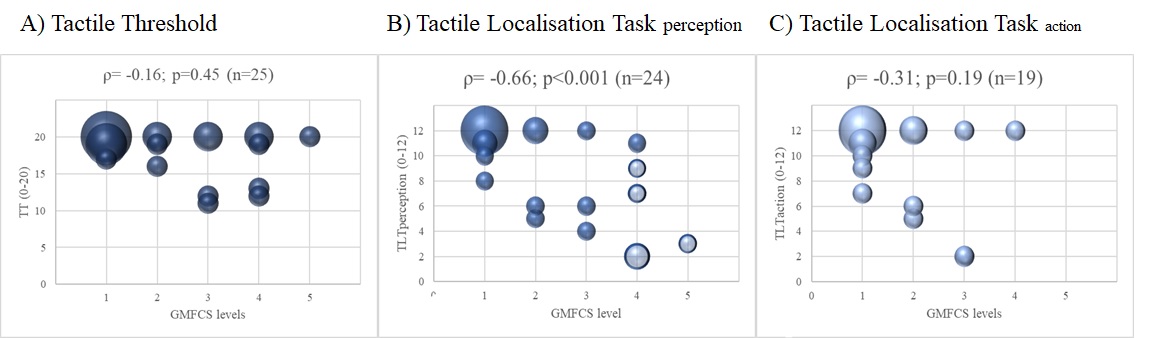

Supplement: Supplementary Figure S2 — Correlations between somatosensory function and the level of gross motor function classification system of the children with cerebral palsy, with spearman correlations (ρ) of the less affected leg. The four shaded dots in B) Tactile Localisation Task (TLT) perception represent the children that could not perform the Tactile Localisation Task (TLT) action. The size of the dots indicates the number of participants (the larger the dots, the more children were pictured). [file Image2.jpeg]
